# Effect of Increasing Maximal Aerobic Exercise on Serum Muscles Enzymes in Professional Field Hockey Players

**DOI:** 10.5539/gjhs.v7n3p69

**Published:** 2014-11-05

**Authors:** Muhsin Hazar, Aynur Otağ, İlhan Otağ, Mehmet Sezen, Ozan Sever

**Affiliations:** 1 Gazi Üniversity, School of Physical Education and Sports, Ankara, Turkey; 2 Cumhuriyet Üniversity, Health Sciences Faculty, Department of Physical Therapy and Rehabilitation, Sivas, Turkey; 3 Cumhuriyet Üniversity, Vocational School of Health Services, Sivas, Turkey

**Keywords:** aerobic exercise, creatine kinase, muscle damage

## Abstract

**Background and Objectives:**

Exercise results in oxidative enzyme increase and micro-injuries in skeletal muscles. The aim of this study was to investigate the effect of maximal aerobic exercise on serum muscle enzymes in professional field hockey players.

This study aims to determine the effect of increasing maximal aerobic exercise on creatine kinase (CK), creatine kinase-MB (CK-MB), aspartate aminotransferase (AST) and alanine aminotransferase (ALT) serum levels.

**Material and Methods:**

31 young professional field hockey players (13 female and 18 male players) volunteered for this study. All participants underwent the shuttle run test. Blood samples were taken from each participant before the shuttle run test. Post test blood samples were taken immediately after exercise and one hour after respectively. Pre and post test CK, CK-MB, AST and ALT values were measured by means of auto analyzer using original kits.

**Results:**

The acute post test measure of the CK level increased in male (p=0.002) and female (p=0.00) sportsmen. CK-MB values obtained one hour after the exercise was lower than those before the exercise in males (p=0.02). In females (p=0.017) and males (p=0.05) AST activity significantly increased immediately after exercise and decreased to resting activity 1 h recovery. ALT significantly increased immediately after exercise in female (p=0.03) and male (p=0.00) athletes and after 1 h recovery ALT activities decreased below resting values.

**Conclusion:**

The timing and severity of exercise used in our study increased CK values, decreased CK-MB values and AST, ALT values increased in female and male field hockey players.

## 1. Introduction

Exercise causes micro-injuries in skeletal muscle. In response to these injuries, several substances are secreted in structure of protein and enzyme. Among these substances, there are muscle injury markers. Hard exercises cause injuries in the structure of skeletal muscle cells, sarcolemma and Z discs (Baird, Graham, Baker, & Bickerstaff, 2012). The micro injuries occurred the level of some enzymes which is revealed by increases in blood (Howatson & Someren, 2008). Creatine kinase (CK) is one of these enzymes showing the injury relating to the muscle (Cruzat, Rogero, Borges, & Tirapegui, 2007; Mckune, Semple, & Peters-Future, 2012). On the other hand, CK-MB is an ISO-form of creatine kinase increasing, especially in myocardial infarction (Brancaccio, Maffulli, & Limongelli, 2007). CK-MB increases during hard exercises, rhabdomyolysis, trauma, myocyte and muscle dystrophy. CK-MB levels reach peak levels in myocard-related infarct, in the serum 4–6 hours after muscle injuries, and remain at high levels during 24 hours (Jois-Bilowich & Tyndall, 2010). Total CK is recognized as non specific while CK-MB is the most specific at the myocardial infarction (Hemalatha, UmaMaheswar, Krithiga, Sankaranarayanan, & Puvanakrishnan, 2013). Aspartate amino transferase (AST) and alanin aminotransferase (ALT) are the indices of cellular necrosis and tissue damage in skeletal muscle (Brancaccio et al., 2007; Duman & Erden, 2014). ALT also known as associated with an increased risk of hepatocellular carcinoma, viral hepatitis and toxic liver necrosis. AST serum level increase in severe damage to the liver (Hemalatha et al., 2013). The increase in AST level is in proportion to the level of cell injury of the body and for this reason, it is an important serum monitoring marker with the following of injury development or recovery period (Karaçalıoğlu, 2007; Nie, Tong, George, Fu, Lin, & Shi, 2011).

Participation in sports is associated with risks of skeletal muscle injuries (Brancaccio et al., 2007). Field hockey is a sport with a long history that has undergone quite a rapid and radical change within the past decade. The advent of the synthetic playing surface has changed the technical, tactical and physiological requirements of the game at all levels, but in particular at the elite level (Manna, Khanna, & Dhara, 2010). There are very few publications in the literature about the muscle damage of field hockey players.

For this reason, our study aims to determine the effect of maximal exercise on CK, CK-MB, AST and ALT serum levels, and to detect the injuries of skeletal and heart muscles that may occur in elite field hockey players taking serum enzyme levels into consideration.

## 2. Material and Methods

### 2.1 Subjects

Professional young female grass hockey players (13) and professional young male grass hockey players (18) attended the study after their is written attendance forms and health reports had been taken (Table 1). The experimental protocol in this study was approved by the local ethics committee at Gazi University, Ankara, Turkey. All subjects were informed about the purpose and risks of the study before signing a written consent. Studies were performed according to the Declaration of Helsinki.

**Table 1:** The Subjects Characteristics

Variables	Female	Male	P
**Age (years)**	18.76±1.09	19.72±1.17	0.03
**Height (cm)**	166.30±4.98	178.38±6.68	0.00
**Weight (kg)**	57.07±5.37	70.83±7.75	0.00
**Sports age (years)**	7.48±1.81	7.33±1.49	0.39

### 2.2 Exercise Protocol

The subjects were applied shuttle run test for it is a method commonly performed. The subjects ran between two lines taking, apart from each other with a distance of 20 meters; the test was started with a slow running of (8km/h). The run started with the first signal sound. Then, the same distance was run again and the second run started. Running speed was adjusted with the signals increasing 0.5 km/h per minute. The test was stopped when the subject failed to follow the set pace of the “beeps” for two successive shuttles, or stopped voluntarily (Rambsbottom, Brewwer, & Williams, 1988). All subjects completed the test was successful. The subjects were instructed not to eat anything three hours before the exercise and not to do sport starting from two days before the exercise and these conditions were taken under control in this way.

### 2.3 Collection of Samples

Blood samples were taken from each participant before exercise, post test blood samples were taken immediately after exercise and one hour after respectively. The authors of this manuscript’s carried out the blood sampling and testing. The serums were kept at -80 °C until the analysis. CK, CK-MB, AST and ALT values were measured by means of auto analyzer using original kits (Roche Hitachi Modular DP Systems, Manheim Germany) at the laboratories of Gazi university medical faculty.

### 2.4 Statistical Analysis

All values were transferred into SPSS 20.0 package, a computer program. The repeated measure ANOVA was used to compare CK, CK-MB, AST and ALT serum levels at measured baseline, immediately and 1-hour after the test. The independent t - test was to compare values between male and female participants. Alpha level was set at p<0.05.

## 3. Results

Subject characteristics shown in Table 1. There is no significant difference in the general characteristics between male and female except sports age.

CK activity significantly increased immediately after the exercise in male (p=0.002) and female sportsmen (p=0.00) İn female and male after 1 h recovery CK activity returned to resting activity. In males, the values one hour after the exercise are lower than the values before the exercise (Table 2).

**Table 2:** Repeated measure of ANOVA comparison of pre and post (immediately and 1-hour after) shuttle run test effect on serum muscle enzyme

Variable	PE	PSE	P1HSE	F-ratio	p-value
	X±S.D	X±S.D	X±S.D		
All participant					
CK/UL	364.29±214.08	393.09±222.2	350.51±187.09	11.56	0.00
CKMB	16.58±10.27	16.87±10.14	14±8.16	3.03	0.056
AST	33.12±24.60	34.32±25.27	30.58±22.83	4.31	0.01
ALT	13.83±7.55	15.38±8.44	12.54±7.22	21.33	0.00
Male only					
CK/UL	437.27±49.16	469.16±50.79	412.16±41.55	7.6	0.002
CKMB	18.61±12.72	16.33±12.63	14.16±10.03	3.93	0.029
AST	40±30.38	40.16±31.56	35.61±28.84	3.21	0.052
ALT	16.83±8.35	19.11±9.22	15.94±7.61	16.66	0.00
Female only					
CK/UL	263.23±184.07	287.76±192.24	265.15±172.85	11.01	0.00
CKMB	13.61±4.45	17.61±5.47	13.76±4.91	2.51	0.10
AST	23±5.99	5.61±6.66	23.61±5.90	4.87	0.017
ALT	9.69±3.4	10.23±2.86	7.84±2.67	7.32	0.03

As for CK-MB values, decreased in both genders after 1 h recovery. The values 1 h after the exercise are lower than the values before the exercise in males and females (p=0.02) (Table 2).

In female AST activity significantly increased immediately after exercise and decreased to resting activity 1 h recovery (p=0.017). No significant changes in AST activities were indicated in men immediately after exercise. On the other hand, there was the significant decrease of AST activity after 1 h recovery in male subjects (p=0.05) (Table 2).

ALT activities significantly increased immediately after exercise in females (p=0.03) and men (p=0.00). In males and females after 1 h recovery ALT activities decreased below resting activities (Table 2).

**Figure 1:**
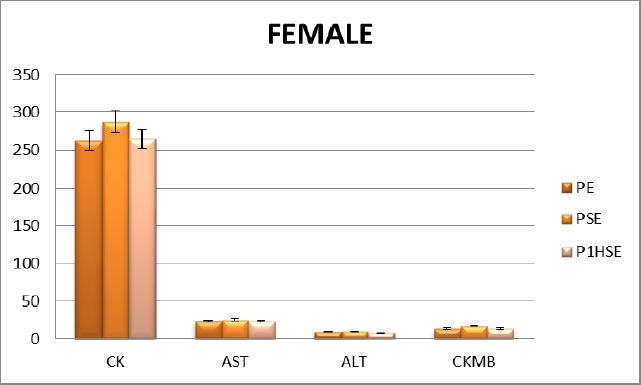
Activities of choosing enzymes after exercise of female field hockey players. (PE, before the exercise; PSE, immediately after the exercise; P1HSE, 1 h recovery activities of enzymes)

**Figure 2:**
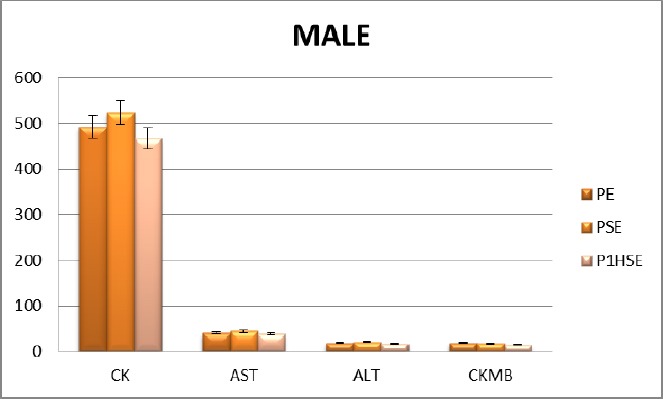
Activities of choosing enzymes after exercise of male field hockey players. (PE, before the exercise; PSE, immediately after the exercise; P1HSE, 1 h recovery activities of enzymes)

When female and male sportsmen are compared, CK values are higher in males all along the study. In terms of CK-MB values, no significant difference is found between the two genders. AST and ALT values are higher in males all along the study (Table 2).

## 4. Discussion

This objective of the study to determine the effect of maximal exercise on CK, CK-MB, AST and ALT serum levels. There are very few publications in the literature about the muscle damage of field hockey players. Therefore, our study may be important. Creatine kinase is a significant enzyme showing muscle injury. Many researchers have stated that CK level increases after exercise (Chen & Hsıeh, 2001; Dousset et al., 2007; Hazar, 2011; Halson, Lancaster, Jeukendrup, & Gleeson, 2003; Nie et al., 2011; Nosaka & Clarkson, 1996; A. Otağ, Deveci, İ. Otağ, & Koşar, 2013; Zembron-Lacny, Slowinska, & Ziemba, 2010). Nie et al found in their study that CK levels were higher all along 24 hours in 12 male runners after a running program of 21 hours (Nie et al., 2011). Types by applying the exercise change CK level. Eccentric-resistance exercises contribute to the increase in CK level more than the other exercises (Baird et al., 2012; Mckune, Semple, & Peters-Future, 2012). This study finding on increased CK level, both in male and female sportsmen after the exercise. Serum CK values were lower in females than males all along the study. This lower level may due to the fact that less enzymes transfer from the muscle to the serum as estrogen hormone decreases membrane permeability after the exercise (Brancaccio et al., 2007).

CK-MB is an ISO-enzyme in heart muscle (Baird et al., 2012). Cardiac cells are an important resource for CK-MB. The value of CK-MB in serum has generally increased after a cardiac injury (Jois-Bilowich & Tyndall, 2010). In the study carried by Nie et al., CK-MB level increased in the serum after a run of 21 km, and it remained high all along 24 hours (Nie et al., 2011). This study found CK-MB values, decreased in both genders after 1 h recovery. It is known that it is slower to put CK-MB out of the blood in sportsmen than cardiac patients (Apple, 1984).

AST and ALT enzymes increase due to muscle injury (Brancaccio et al., 2007; Levinger, Goodman, Peake, Granham, Jerums, & Selig, 2009; Nie et al., 2011). AST is an enzyme which has been used to make a diagnosis in heart muscle injuries recently (Jois-Bilowich & Tyndall, 2010). Cordova et al, made a study with volleyball players through one season, and AST and ALT values were found higher than non-sportsmen after the training (Cordova, Sureda, Tur, & Pons, 2010). Harbili et al found no difference in ALT and AST values after the exercise in their study (Harbili, Gencer, Ersöz, & Demirel, 2008). Similarly, Hazar et al did not find any difference in AST values after the exercise in their study carried out with professional sportsmen (S. Hazar, M. Hazar, Korkmaz, Bayil, & Gürkan, 2011). This study found, AST values increased in elite female sportsmen after the exercise, and then returned to their former levels one hour after the exercise. On the other hand, in males, AST values were found lower than their former levels one hour after the exercise. ALT values increased after maximal aerobic exercise in both female and male elite sportsmen, and then they were found lower than their former levels one hour after the exercise. This study finding on changed AST and ALT values in both female and male sportsmen.

## 5. Conclusion

The timing and severity of exercise used in this study increased CK values, decreased CK-MB values and AST, ALT values increased in female and male elite field hockey players.
